# Attentional sensitization of unconscious visual processing: Top-down influences on masked priming

**DOI:** 10.2478/v10053-008-0102-4

**Published:** 2012-02-15

**Authors:** Markus Kiefer, Sarah C. Adams, Monika Zovko

**Affiliations:** Department of Psychiatry, University of Ulm, Germany

**Keywords:** automatic processes, unconscious visual processing, attentional control, semantic priming, visuo-motor priming, subliminal perception, consciousness

## Abstract

Classical theories of automaticity assume that automatic processes elicited by unconscious stimuli are autonomous and independent of higher-level cognitive influences. In contrast to these classical conceptions, we argue that automatic processing depends on attentional amplification of task-congruent processing pathways and propose an attentional sensitization model of unconscious visual processing: According to this model, unconscious visual processing is automatic in the sense that it is initiated without deliberate intention. However, unconscious visual processing is susceptible to attentional top-down control and is only elicited if the cognitive system is configured accordingly. In this article, we describe our attentional sensitization model and review recent evidence demonstrating attentional influences on subliminal priming, a prototypical example of an automatic process. We show that subliminal priming (a) depends on attentional resources, (b) is susceptible to stimulus expectations, (c) is influenced by action intentions, and (d) is modulated by task sets. These data suggest that attention enhances or attenuates unconscious visual processes in congruency with attentional task representations similar to conscious perception. We argue that seemingly paradoxical, hitherto unexplained findings regarding the automaticity of the underlying processes in many cognitive domains can be easily accommodated by our attentional sensitization model. We conclude this review with a discussion of future research questions regar-ding the nature of attentional control of unconscious visual processing.

## Introduction

Unconscious automatic processes are traditionally thought to occur autonomously and independently of top-down control (Posner & Snyder, 1975; Schneider & Shiffrin, 1977). According to classical theories, automatic processes (a) are independent of capacity-limited attentional resources, (b) are not prone to interference from other processes, (c) can act in parallel, and (d) are unconscious (Posner & Snyder, 1975; Schneider & Shiffrin, 1977). Top-down control by attention, action goals, and task sets is assumed to be restricted to processes that are conscious.

Although lacking direct empirical support, this classical view is implicit in current theorizing about automaticity and strongly influences contemporary conceptions of cognitive control: Based upon the assumption that automatic processes are autonomous, a behavioral or neurophysiological effect has to be invariant in order to index a “truly automatic” process (Pessoa, Kastner, & Ungerleider, 2003). Such operational definitions of *automaticity*, which are essentially influenced by the classical view, can be found in many areas of psychology and neuroscience such as in object or face recognition (e.g., Pessoa, McKenna, Gutierrez, & Ungerleider, 2002; Wiese, Schweinberger, & Neumann, 2008), action preparation (e.g., Bub & Masson, 2010), and emotional processing (e.g., Pessoa et al., 2002). Given that attention and task demands are frequently found to modulate behavioral and neurophysiological effects, it is difficult to identify processes that actually meet the classical criteria for automaticity. The apparent lack of processes that fully meet the criteria of automaticity renders the classical view of automaticity unsatisfactory (see also Moors & De Houwer, 2006). Furthermore, the classical stance of automaticity implies a considerable inflexibility of the cognitive system: Conscious goal-directed information processing would be massively influenced by various unconscious processes. Such inflexibility would place tremendous demands on conscious control, because the intended action could only be ensured by inhibiting numerous interfering response tendencies (Botvinick, Braver, Barch, Carter, & Cohen, 2001).

A number of recently refined theories of automaticity allows for more flexibility and adaptability of automatic processing and unconscious cognition (Kiefer, 2007; Kiefer & Martens, 2010; Moors & De Houwer, 2006; Naccache, Blandin, & Dehaene, 2002; Neumann, 1990). These theories converge on the assumption that the cognitive system has to be configured by attention and task sets in order for automatic processes to occur. For instance, the theory of direct para-meter specification (DPS) by Neumann (1990) posits that unconscious information will only be processed and influence the motor response to a target stimulus to the extent that it matches current intentions. Unlike classical theories, refined theories assume that automatic processes are critically dependent on higher-level, top-down factors such as attention, intentions, and task sets that orchestrate the processing streams toward greater optimization of task performance. Given this dependency on the precise configuration of the cognitive system, one might as well speak of *conditional automaticity* (Bargh, 1989; Logan, 1989).

### Subliminal priming by masked stimuli

Typical examples of unconscious automatic processes are subliminal priming (e.g., facilitatory) effects elicited by masked visual stimuli that are not consciously perceived. Different forms of priming can be distinguished, depending on the relation between prime and target (Kiefer, 2007). Masked visuo-motor response priming denotes faster responses to visual shape targets, when the masked prime (also a visual shape) indicates the same rather than a different response (Ansorge & Neumann, 2005; Vorberg, Mattler, Heinecke, Schmidt, & Schwarzbach, 2003). This form of priming depends on visuo-motor processes giving rise to response conflict. Masked semantic priming, in contrast, reflects access to word meaning (Carr & Dagenbach, 1990; Kiefer, 2002; Kiefer & Spitzer, 2000). It denotes facilitation of a response to a target word, when it is preceded by a semantically related masked prime word (e.g., *chair-table*). In line with the assumption that priming is mediated by different processes, different forms of priming activate distinct brain regions: Visuo-motor response priming recruits occipito-parietal areas (Wolbers et al., 2006) known to be involved in visual form processing (ventral pathway) as well as in object grasping and motor preparation (dorsal pathway). Semantic priming depends on anterior temporal areas (ventral pathways) supporting semantic integration (Kiefer & Pulvermüller, 2011; Nobre & McCarthy, 1995).

As a complement to behavioral priming effects, event-related potentials (ERPs) recorded from the scalp capture task-specific priming processes on-line during task performance. Semantic priming modulates the N400 ERP component, a negative deflection peaking at about 400 ms with a centro-parietal topography (Kutas & Hillyard, 1980). The N400 semantic priming effect is reflected by an attenuated N400 amplitude (i.e., relatively less negative voltage) to a target when preceded by a semantically related as compared with an unrelated prime (Bentin, McCarthy, & Wood, 1985; Kiefer, Weisbrod, Kern, Maier, & Spitzer, 1998). Intracranial ERP recordings (Nobre & McCarthy, 1995) and source analyses of scalp potentials (Kiefer, Schuch, Schenck, & Fiedler, 2007) have implicated a region in the anterior-medial temporal lobe in generating the N400 ERP component. Visuo-motor response priming, in contrast, modulates ERPs over the occipito-parietal scalp in a time window between 200-400 ms (Jaśkowski, Skalska, & Verleger, 2003; Martens, Ansorge, & Kiefer 2011). These ERPs most likely arise from the parietal visuo-motor system as identified in a previous functional magnetic resonance imaging (fMRI) study (Wolbers et al., 2006). In contrast to behavioral measures, which reflect the output of the entire processing chain, ERPs have the advantage of directly capturing cognitive processes online during task performance.

Before we review the latest findings demonstrating attentional influences on subliminal priming as an example of an automatic process, we introduce our theoretical view of attentional control of unconscious visual processing in the next section.

## The attentional sensitization model for top-down control of unconscious cognition

Although refined theories of automaticity converge on the assumption that automatic processes are susceptible to top-down control, there is as yet no general theoretical framework that accounts for a number of top-down factors and different forms of automatic processes. We have therefore recently developed the attentional sensitization model of unconscious cognition (Kiefer & Martens, 2010) that aims at explaining the various influences of top-down attention on different forms of unconscious automatic processing. According to this model, attentional influences originating from task sets enhance task-relevant unconscious processes while attenuating task-irrelevant unconscious processes. Much as conscious perception is influenced by attentional mechanisms, unconscious cognition is assumed to be controlled by top-down signals from prefrontal cortex (Haynes et al., 2007) that increase or decrease the sensitivity of processing pathways for incoming sensory input (Bode & Haynes, 2008; Hopfinger, Buonocore, & Mangun, 2000; Hopfinger, Woldorff, Fletcher, & Mangun, 2001). Processing in task-relevant pathways is enhanced by increasing the gain of the neurons in the corresponding areas, whereas processing in task-irrelevant pathways is attenuated by a decrease of the gain (Reynolds, Pasternak, & Desimone, 2000). *Gain* is a parameter in neural network modeling, which influences the probability that a neuron fires at a given activation level (Hamker, 2005). Single cell recordings in non-human primates have shown that the likelihood of a neuron firing, given a constant sensory input, is enhanced when the stimulus dimension that is preferentially processed by the neuron is attended to (e.g., Treue & Martínez-Trujillo, 1999). We thus assume that an attentional sensitizing mechanism gradually enhances and attenuates stimulus processing irrespective of whether the stimulus is consciously perceived or not (Kiefer & Martens, 2010).

The attentional sensitization model suggests that, in a manner similar to controlled processes, automatic processes (a) should depend on available attentional resources, and (b) are susceptible to top-down control by currently active task representations. Attentional sensitization of automatic processing by task representations is achieved by enhancing the sensitivity of task-relevant pathways and by attenuating the sensitivity of task-irrelevant pathways.

Although attentional top-down control of both unconscious and conscious cognition shares basic computational principles, top-down control for conscious processing is more flexible. For this reason, we distinguish between *preemptive* and *reactive control* (Ansorge, Fuchs, Khalid, & Kunde, 2011; Ansorge & Horstmann, 2007; Ansorge, Kiss, & Eimer, 2009; Kiefer, 2007; Kiefer & Martens, 2010). In *preemptive control*, top-down influences are initiated in advance of stimulus pre-sentation. Preemptive control can be exerted for both conscious and unconscious stimulus presentation, whereas only consciously perceived stimuli are susceptible to reactive control in response to ongoing or completed stimulus processing. For that reason, subliminal information cannot be used for determining further strategic processing steps in a deliberate fashion (Merikle, Joordens, & Stolz, 1995). This means that top-down control of unconscious cognition must occur implicitly on the grounds of currently activated action goals or the consciously perceived outcome of overt behavior. As a consequence, intentional application of control and on-line modification is restricted to conscious processes (Dehaene, Changeux, Naccache, Sackur, & Sergent, 2006). Finally, attentional influences on unconscious cognition are presumably facilitatory, that is, they depend on differential attentional sensitization, whereas active inhibition of task-irrelevant information appears to be confined to controlled processing of consciously perceived stimuli (Merikle et al., 1995; Neely, 1977; Posner & Snyder, 1975). Thus, according to the attentional sensitization model (Kiefer & Martens, 2010), conscious “strategic” stimulus processing allows for a greater adaptability and flexibility of top-down control than “automatic” processing under unconscious viewing conditions.

In the upcoming two parts of this article, we will review latest evidence demonstrating a variety of attentional influences on several forms of unconscious priming. In the next section, we describe findings that support our notion of attentional sensitization of unconscious visual processing although these studies were not specifically designed to test our model. Empirical work that specifically aims at testing the attentional sensitization model using the induction task paradigm is discussed in detail in the subsequent section, *Specifying attentional influences on subliminal priming with the induction task paradigm*.

## Attentional influences on subliminal priming

Although the classical view of automaticity is prevailing and still dominates current research, evidence for attentional top-down control of unconscious visual processing has been accumulated during the last years. Several attentional manipulations have been shown to reliably modulate subliminal priming effects. This highlights the generality and robustness of attentional effects on unconscious visual processing. In this section, we review findings from studies demonstrating that subliminal priming (a) depends on attentional resources, (b) is susceptible to stimulus expectations, and (c) is influenced by action intentions.

### Influence of attentional resources

Unconscious priming has been shown to depend on attentional top-down amplification and attentional resources similar to conscious visual perception: In a masked semantic priming study (Kiefer & Brendel, 2006), an attentional cue was presented that prompted participants to attend to the stimulation stream either during the time window of masked prime presentation or already 1 s earlier. In the latter long cue-prime interval condition, subjects disengaged attention when the masked prime was finally presented. Kiefer and Brendel obtained a semantic priming effect on the N400 ERP component, but only when the masked prime was presented within the time window of attention. In a similar study, masked response priming was only obtained when the onset of the prime-target pairs was temporally predictable and therefore attended to (Naccache et al., 2002). Furthermore, masked semantic priming was significantly reduced when the masked prime was preceded by a difficult task requiring greater attentional resources compared with an attentionally undemanding task (Martens & Kiefer, 2009). In addition to temporal attention and attentional resources, unconscious visual processing depends on spatial attention: In patients with blindsight, spatial cueing was found to improve discrimination performance without awareness (Kentridge, Heywood, & Weiskrantz, 1999, 2004) suggesting that unconscious visual processing benefits from spatial attentional amplification comparable to conscious visual perception (Posner, Snyder, & Davidson, 1980). These findings are in line with our proposal (Kiefer & Martens, 2010) that attention and conscious experience are functionally independent to some extent and should not be equated (see also Koch & Tsuchiya, 2007; Van Boxtel, Tsuchiya, & Koch, 2010).

### Influence of stimulus expectations

Subliminal processing of stimuli strongly depends on stimulus expectations that include what kind of stimulus is likely to occur within a given situation. Expected subliminal stimuli receive attentional amplification and are further processed whereas processing of unexpected stimuli is attenuated (Eckstein & Perrig, 2007; Kiesel, Kunde, Pohl, Berner, & Hoffmann, 2009; Kunde, Kiesel, & Hoffmann, 2003). Of course, these attentional expectations cannot be established by unconsciously presented stimuli themselves, but are formed by consciously perceived stimuli presented in a specific situation, for instance, by the visible target stimuli of a priming paradigm. It has been shown that the nature of visible target stimuli included in an experiment strongly influences subliminal priming effects. This phenomenon has been mostly shown within the domain of response priming: Masked stimuli prime responses only if they are expected and represent possible release conditions for prepared actions to the visible targets (Eckstein & Perrig, 2007; Kiesel et al., 2009; Kunde et al., 2003). For instance, subliminal response priming effects elicited by novel primes, which are not presented as targets, are only obtained when they belong to or are at least similar to the attentional set established by the visible targets. Kunde and colleagues (2003) showed that subliminally presented numbers prime numerical categorizations of visible numbers only when they are located within the magnitude space spanned by the visible targets (e.g., the prime numbers “2” and “3” are within the magnitude space spanned by the visible targets “1” and “4”), but not when they are outside the magnitude space spanned by the visible targets (e.g., the prime numbers “1” and “2” are outside the magnitude space spanned by the visible targets “3” and “4”). Similar expectancy effects on response priming have been observed for verbal stimuli within a semantic categorization task when the target set size was manipulated (Kiesel, Kunde, Pohl, & Hoffmann, 2006). In one condition, target set size was large (40 targets) so that a variety of words from different semantic categories was expected. In the other condition, target set size was small (four targets) so that attention could be focused on a narrow set of stimuli. In line with the assumption that stimulus expectations influence processing of subliminal information, response priming for novel subliminal prime words was only obtained for the large, but not for the small target set. These findings demonstrate that the content of an attentional set establishes stimulus expectations that sensitize processing pathways for expected stimuli even when they remain unconscious. As a result, these expected subliminal stimuli elicit priming effects. In a similar vein, stimulus expectations that are based on image statistics associated with specific object categories (such as animals vs. tools) can influence unconscious gaze control (Li, VanRullen, Koch, & Perona, 2002; Torralba & Oliva, 2003).

### Influence of action intentions

Masked response priming has been shown to depend on action intentions: Ansorge and colleagues (Ansorge, Heumann, & Scharlau, 2002; Ansorge & Neumann, 2005) found that unconsciously perceived masked primes trigger responses only if they are congruent with the current intentions of a person. Response priming effects were abo-lished when task instructions were changed in such a way that primes ceased to be task-relevant. For instance, primes and targets with a similar shape elicited subliminal response priming effects (i.e. faster response for primes and targets with similar shapes) only when the response decision was based on the target’s shape (Ansorge & Neumann, 2005). However, when the instruction of the target task was changed such that the response decision was based on the target’s color, response priming effects disappeared although primes and targets still exhibited similar or dissimilar shapes (Ansorge & Neumann, 2005). In a comparable experiment, shape or color congruency of masked primes and visible targets only primed target responses, when the corresponding prime feature (e.g., shape during shape decisions on the target) was relevant in the target task (Tapia, Breitmeyer, & Shooner, 2010). The task-irrelevant prime feature did not influence responses to targets.

In a continuation of this line of research, the capture of visuo-spatial attention by unconscious stimuli likewise was shown to depend on the match between stimulus features and a top-down search template directed towards the task-relevant visual features of the targets (Ansorge, Horstmann, & Worschech, 2010; Ansorge et al., 2009; Held, Ansorge, & Müller, 2010). Top-down effects on attentional capture by unconscious stimuli were discussed in detail by Ansorge, Horstmann, and Scharlau (2011) and by Reuss, Pohl, Kiesel, and Kunde (2011).

Modulatory effects of action intentions have also been observed on subliminal processing of semantic word meaning: During semantic categorizations of target words (evaluative valence decision vs. animacy decision), affective (positive vs. negative valence) or animacy (living vs. non-living) congruency of preceding subliminally presented prime words elicited only priming effects on the target decision when the corresponding meaning dimension was also task-relevant in the target task (Eckstein & Perrig, 2007). Similarly, spatial congruency of prime and target words indicating either an elevated (e.g., *above*) or a lowered location (e.g., *below*) produced priming effects only during a spatial target task, but not during a target task with numbers of high and low numerical magnitude (Ansorge, Kiefer, Khalid, Grassl, & König, 2010). These findings suggest that action intentions sensitize congruent and desensitize incongruent unconscious processing pathways: We propose that an attentional top-down signal enhances unconscious processing of the stimulus dimension that matches the current intention. This attentional sensitization mechanism results in subliminal priming effects on responses to visible targets only for stimulus dimensions that are congruent with the current action intention. Although action intentions apparently attenuate the processing of task-irrelevant subliminal information, this does not preclude the possibility that task-irrelevant stimulus dimensions can involuntarily influence task-relevant responses when they partially match with the action intention and thus belong to the currently active task set to some extent. Such phenomena are typically observed in interference paradigms with visible stimuli. For instance, naming the ink color of a color word in the Stroop task (Stroop, 1935) receives interference by the irrelevant meaning of the color word. Similarly, classification reactions with the left or right hands to visual stimuli are influenced by their irrelevant spatial position as observed in the Simon task (Simon, 1990). Interestingly, in line with our attentional sensitization model, these interference effects reflecting automatic processing of irrelevant stimulus dimension can be abolished if the attentional set is changed such that the partial match of the task-irrelevant stimulus dimension with the current action intention is removed (Raz, Kirsch, Pollard, & Nitkin-Kaner, 2006).

## Specifying attentional influences on subliminal priming with the induction task paradigm

Unconscious priming does not only depend on action intentions, but also on task sets, which are active during the presentation of the masked prime (Kiefer, 2007; Kiefer & Martens, 2010; Martens et al., 2011). Similar to intentions, task sets are assumed to trigger an attentional sensitization mechanism that enhances processes in task-congruent pathways while attenuating task-incongruent processes. In this section, we review results of recent studies with the induction task paradigm that allows specifying attentional influences originating from task sets on various forms of unconscious visual processing at a fine-grained level. The induction task paradigm has been developed to test specific predictions of the attentional sensitization model, but can be generally used to identify the influence of task sets on conscious or unconscious visual perception.

In line with earlier proposals (Rogers & Monsell, 1995), we define *task sets* as an adaptive configuration of the cognitive system which is necessary to efficiently perform a given task (see also Kiesel et al., in press). The concept of task set is related to that of intention, but is more specific because it only refers to the immediate computational consequences of pursuing a current goal during task performance that determine the configuration of the cognitive system. The concept of intention is broader because it additionally includes the conscious representation of the goal and the subjective state of commitment to perform a goal-related action (Ansorge & Neumann, 2005; Goschke, 2002).

**Figure 1.**
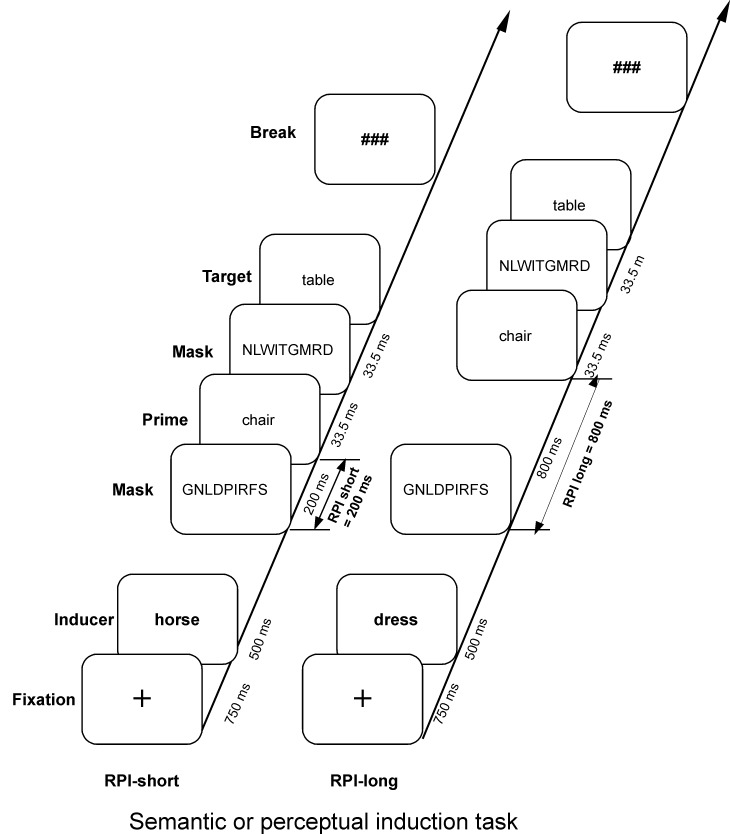
Temporal sequence of one trial in the semantic and perceptual induction task conditions. The masked prime word was presen-ted either 200 ms or 800 ms following the response to the induction task (response stimulus interval, RPI) that is intended to elicit the corresponding task set. The semantic induction task required semantic classification (forced choice living/non-living decision) to the inducing word, whereas the perceptual induction task required a forced choice perceptual classification decision of the first and the last letter (open/closed shape) of the inducing word. Modified after “Attentional Sensitization of Unconscious Cognition: Task Sets Modulate Subsequent Masked Semantic Priming” by M. Kiefer and U. Martens (2010), *Journal of Experimental Psychology: General, 139*, pp. 464-489.

In order to determine attentional top-down influences of task sets on unconscious semantic and visuo-motor response priming, we have recently developed an induction task paradigm (cf. Figure 1) that exploits the temporal dynamics of task set activation (Kiefer & Martens, 2010). Consider a scenario in which the subject needs to perform two tasks in quick succession, the second task being a subliminally primed decision task preceded by a semantic or a perceptual classification task. According to the attentional sensitization model, these previously performed tasks should differentially influence the subsequent masked priming effect (Kiefer & Martens, 2010).

In our induction task paradigm, prior to the masked priming procedure, participants were engaged in different induction tasks (e.g., semantic classification vs. perceptual classification) designed to induce a specific task set (e.g., semantic or perceptual task set). The different induction tasks were presented in separate blocks in order to avoid task switching effects between trials with different induction tasks. The induction tasks were followed immediately by a primed decision task (e.g., lexical decision for semantic priming or shape decision for visuo-motor priming). Across experiments, we systematically varied the type of induction task to specify the attentional mechanisms which enhance or attenuate unconscious processing. According to the proposed attentional sensitization model, task representations enhance and attenuate processing streams in order to facilitate processing in congruency with higher-level goals: Automatic processes that match task representations are assumed to be amplified, while other automatic processes should be attenuated.

### Influence of perceptual and semantic induction tasks on subliminal semantic and visuo-motor priming

Using the induction task paradigm, we systematically investigated the influence of previously performed tasks on subsequent masked semantic priming within a lexical decision task (word/non-word decision) in three experiments (Kiefer & Martens, 2010). We asked whether a semantic task set induced by a semantic decision task (induction task) immediately before masked prime presentation sensitizes semantic processing pathways and enhances subliminal semantic priming. In contrast, a perceptual task set induced by a task that requires attention to visual stimulus features was assumed to desensitize semantic pathways and therefore to attenuate subsequent subliminal semantic priming. We varied the time interval (either 200 or 800 ms) between the response to the induction task and the onset of the prime (RPI) in order to obtain information on how the influence of the induction task on masked priming unfolds over time. We expected that a semantic induction task would sensitize semantic processing pathways and thus would enhance semantic priming only at the short RPI (200 ms) because the task switching literature suggests that a task representation is active for about 600 ms after task completion (Rogers & Monsell, 1995), but is actively inhibited thereafter (Mayr & Keele, 2000). In all experiments, subliminal priming effects were assessed with behavioral (shorter reaction time [RT] to semantically related than to unrelated prime target pairs) and ERP measures (larger amplitude of the N400 ERP component, an electrophysiological index of semantic processing, for semantically unrelated than for related prime-target pairs).

Across experiments, the difficulty of the semantic and perceptual induction tasks as well as their verbal or non-verbal nature was systematically varied. In Experiment 1, participants performed an easy semantic word classification task (living/non-living decision) and a difficult perceptual letter classification task with words (deciding whether first or last letter of a word has a closed or open shape). In Experi-ment 2, the difficulty of the induction tasks was reversed: difficult semantic word classification (deciding whether a word refers to something dry or wet) versus easy perceptual letter classification (deciding whether first or last letter of a word is the letter t). In Experiment 3, non-verbal induction tasks had the same level of difficulty: semantic classification (living/non-living decision) versus perceptual classification of object pictures (round vs. elongated shape decision). At the short RPI, semantic priming effects on RT and N400 ERP component were obtained when a semantic task set was induced immediately before subliminal prime presentation, whereas a previously induced perceptual task set attenuated priming.

Comparable results were obtained regardless of the difficulty level and the verbal or non-verbal nature of the induction tasks. In line with the attentional sensitization model, unconscious semantic processing is enhanced by a semantic task set and attenuated by a perceptual task set. At the long RPI, significant priming was found after the perceptual induction task, but not after the semantic task. The priming effects at the long RPI suggest that after 800 ms, the task set of the induction task was abandoned and a reconfiguration of the cognitive system in preparation for the upcoming lexical task took place (Kiefer & Martens, 2010): Semantic pathways are sensitized when the perceptual induction task had been abandoned, but they are desensitized when the semantic induction task had been abandoned. This result pattern is compatible with the notion of a backward inhibition mechanism that suppresses irrelevant task sets in preparation of the next task (Houghton, Pritchard, & Grange, 2009; Mayr & Keele, 2000). The differential modulatory effects of induction tasks on masked priming provide a window to the dynamic nature of cognitive reorganization that takes place during task set switching that in turn influences top-down control of unconscious cognition.

In the second study (Martens et al., 2011), we contrasted in two experiments the influence of a perceptual (round vs. elongated object classification) with that of a semantic induction task (living vs. non-living object classification) on masked semantic word priming and visuo-motor response priming of geometrical target shapes. In the visuo-motor priming task, participants performed right or left hand responses to discriminate between geometrical target shapes (e.g., circle or square). The visible target was preceded by a masked prime that indicated either the same or a different motor response, but was never combined with the identical shape to avoid repetition effects. In contrast to semantic priming, visuo-motor response priming modulates occipito-parietal ERP components between 200-500 ms (Jaśkowski et al., 2003). The attentional sensitization model predicts that perceptual and semantic induction tasks differentially modulate these two forms of subliminal priming. In line with our attentional sensitization model (Kiefer & Martens, 2010), behavioral and electrophysiological effects showed a differential modulation of subliminal priming by the induction tasks: As in the previous experiments, semantic priming was found following the semantic but not following the perceptual induction task. Visuo-motor priming, depending on access to visual shape information, was only observed after the perceptual but not after the semantic induction task. Hence, unconscious processes in visuo-motor and semantic processing streams are coordinated in congruency with current task sets.

### Determining the attentional boundary conditions for subliminal semantic and visuo-motor priming

In two further studies, we determined the nature of task sets which boost semantic or visuo-motor priming. In the first study (Adams & Kiefer, results not published yet), we wanted to better characterize the task set that enhances unconscious semantic processing. Previously, we found enhanced subliminal semantic priming following a semantic word classification task, but reduced priming following a perceptual letter classification task (Kiefer & Martens, 2010). Unlike the semantic word classification task, the perceptual letter classification task discouraged word reading and focused attention to single letters. It is therefore an open question whether a phonological task, which involves reading processes, suffices to enhance subsequent semantic priming. It has been suggested that word reading unintentionally includes semantic activation because reading is strongly linked with semantic analysis through numerous practice instances in natural reading situations (Posner & Snyder, 1975).

In two experiments, we varied the nature of the phonological induction task (phonological word vs. phonological letter categorization) to test the boundary conditions for unconscious semantic processing to occur and contrasted them to a semantic induction task. In one experiment, we used a phonological induction task that required attention to the entire word and thus could permit word reading (phonological word induction task). We asked whether this non-semantic task set permits subsequent subliminal semantic priming. In the word induction task, participants had to decide whether words comprised a vowel as first or last letter (e.g., *autumn*, *bottle*, *ocean*) or a consonant as first and as last letter (e.g., *garden*, *paper*). Priming following this phonological induction task was compared with a semantic induction task, in which words had to be classified according to whether they refer to living (e.g., *pilot*, *apple*, *dog*) or non-living objects (e.g., *castle*, *pencil*, *bottle*). We found somewhat smaller subliminal priming effects following the phonological than the semantic induction task although this difference in priming was not significant. In the second experiment, word reading was discouraged in the phonological induction task by presenting words with a capital letter at one position (phonological letter induction task). In this induction task, attention was allocated to phonological aspects of single letters: Participants had to decide whether the capital letter was either a vowel (e.g., *jewEl*, *fAble*) or a consonant (e.g., *oRacle*, *breaTh*). In the semantic induction task, participants again performed a semantic classification. In the latter experiment, semantic priming effects were only observed following the semantic classification, but not following the phonological letter classification induction task. The results of these two experiments show that attention to single letters/phonemes of a word strongly disrupts subsequent semantic processing of unconsciously presented primes (for similar task effects on visible primes, see Maxfield, 1997). An attentional orientation towards word phonology also reduced subsequent subliminal semantic priming, but less pronounced compared with the phonological letter induction task. Dual route models of reading (Coltheart, Curtis, Atkins, & Haller, 1993; Seidenberg & McClelland, 1989) can account for the less pronounced modulation of the subliminal priming effect by the phonological word induction task: According to these models, word reading includes both semantic and non-semantic pathways. Although reading may include implicit access to semantics and can sensitize semantic processing pathways as shown by the semantic priming literature (e.g., Neely, 1991), word reading may bypass semantics in specific conditions (Coltheart et al., 1993; Perry, Ziegler, & Zorzi, 2007; Seidenberg & McClelland, 1989). The two alternative processing pathways underlying word reading may lead to considerable interindividual variability with regard to the specific nature of the phonological task set activated by the phonological word induction task (non-semantic vs. semantic route). This may result in a less reliable reduction of subliminal semantic priming compared with the phonological letter task, which unequivocally activates a non-semantic task and thus clearly desensitizes semantic pathways.

In a continuation of this line of research, we were interested in a fine-grained analysis of perceptual induction task effects on unconscious visuo-motor response priming. There is evidence that shape and color of visible objects can be attended to and processed independently of each other (Boucart, Humphreys, & Lorenceau, 1995). Based on these findings, we varied the induction task within the perceptual domain to further assess whether the proposed attentional sensitization mechanism not only distinguishes between broad cognitive domains such as visual versus semantic stimulus attributes but also specifically sensitizes stimulus attributes within the perceptual domain (Zovko & Kiefer, results not published yet). We contrasted the effects of a shape-decision induction task similar to a previous experiment (Kiefer & Martens, 2010) with a novel color-decision task, in which the hue of colored object pictures had to be classified (red vs. blue hue). In the visuo-motor priming task, participants performed right or left hand responses to discriminate between geometrical target shapes (Martens et al., 2011). We found occipito-parietal ERP priming effects only subsequent to the shape induction task. No such effects were found subsequently to the color induction task. These results show that attentional sensitization of unconscious cognition can also occur within perceptual subdomains, such as shape and color attributes. These attentional influences modulate subliminal visuo-motor response priming very fine-grained at the level of specific visual object features.

These few examples show that the induction task paradigm, combining a task for inducing task sets with a subsequent priming paradigm, could serve as an important tool for elucidating the attentional configuration necessary for certain subliminal processes to occur (e.g., semantic or visuo-motor).

## Attentional sensitization of unconscious visual processing: The controlled nature of automaticity

In the previous sections, we have reviewed recent findings demonstrating attentional influences on unconscious visual processing. Accumulating evidence demonstrates that unconscious visual processing is susceptible to attentional control similar to conscious visual processing: Subliminal priming effects, prototypical examples of automatic processes, are modulated by attentional resources, stimulus expectations, action intentions, and task sets. Hence, in contrast to classical theories of automaticity (Posner & Snyder, 1975; Schneider & Shiffrin, 1977), automatic processes elicited by unconscious visual stimuli are under attentional control to some extent. The findings reviewed here are generally in line with refined theories of automaticity (Moors & De Houwer, 2006; Naccache et al., 2002; Neumann, 1990). They specifically support the notion of attentional sensitization of processing pathways that enhances and attenuates automatic processing elicited by unconsciously perceived stimuli in congruency with task representations (Kiefer, 2007; Kiefer & Martens, 2010). We propose that processing can occur automatically in the sense that it does not depend on conscious awareness and that it is initiated without deliberate intention. However, automatic processing is susceptible to attentional top-down control and is only elicited if the cognitive system is configured accordingly. Thus, unconscious automatic processing and the notion of attentional control is not a contradiction as it has been previously thought (Maxfield, 1997; Pessoa et al., 2002; Posner & Snyder, 1975; Schneider & Shiffrin, 1977).

In the next section, we will show that seemingly paradoxical, hitherto unexplained findings in many cognitive domains regarding the automaticity of the underlying processes can be easily accommodated by our attentional sensitization model.

### Attentional sensitization and the automaticity of cognition and emotion

Within the research of semantic processing, it has been argued that semantic processing is not automatic, but requires controlled access to conceptual meaning (Duscherer & Holender, 2002; Henik, Friedrich, Tzelgov, & Tramer, 1994). This is because semantic priming with consciously perceived stimuli strongly depends on attentional orientation towards the prime word (for a review, see Deacon & Shelley-Tremblay, 2000; Maxfield, 1997). Several studies found reduced or absent semantic priming when the prime word was presented outside the focus of attention (Kellenbach & Michie, 1996; McCarthy & Nobre, 1993) or when participants were required to attend to perceptual letter features of the prime (e.g., a letter search task) and not to its meaning (Chiappe, Smith, & Besner, 1996; Mari-Beffa, Valdes, Cullen, Catena, & Houghton, 2005). These findings are taken as evidence that access to conceptual meaning is confined to a controlled processing mode. However, several other studies demonstrating that unconsciously perceived prime words can elicit semantic priming effects favor the view that semantic processing can also occur in an automatic fashion (Carr & Dagenbach, 1990; Draine & Greenwald, 1998; Kiefer, 2002; Kiefer & Spitzer, 2000; Rolke, Heil, Streb, & Henninghausen, 2001). This apparent contradiction can be easily resolved if one assumes that even automatic processes depend on top-down control through attentional sensitization (Kiefer & Martens, 2010). According to our attentional sensitization model, semantic processing is *automatic* in the sense that it is involuntarily initiated even under unconscious viewing conditions. However, unconscious automatic processes are susceptible to attentional modulation and are not invariantly triggered by the appropriate stimulus in a purely bottom-up fashion.

Similar paradoxical findings regarding the automaticity of pro-cesses have been reported in many other areas of psychology such as sensory-motor preparation (Bub & Masson, 2010), emotion (Pessoa et al., 2003), and cognitive deficits in psychiatric patients (Kiefer, Martens, Weisbrod, Hermle, & Spitzer, 2009). Just to give an example: There is evidence that emotional stimulus information can be processed outside conscious awareness in an automatic fashion (Gaillard et al., 2006; Kemp-Wheeler & Hill, 1992; Morris, Öhman, & Dolan, 1998; Öhman & Soares, 1998). Other findings show, however, that emotional information is only accessed within a strategic processing mode: The typical increase of neural activity to emotional faces in the amygdala, a subcortical structure essentially relevant for assigning emotional arousal to a stimulus, was abolished when a demanding secondary task strongly depleted attentional resources (Pessoa et al., 2002). As emotional brain activity depends on attention, it has been concluded that emotional processing is not automatic (Pessoa et al., 2003). Again, these seemingly discrepant findings of the automaticity of emotional processing can be accommodated in the attentional sensitization model. Our framework assumes that automatic processes, similar to controlled processes, depend on an attentional amplification that sensitizes processing pathways. If a se-condary task depletes attentional resources, the potential of an affective stimulus to automatically trigger an emotional response is reduced or abolished.

These examples demonstrate that the proposed attentional sensitization model applies to many domains and has the explanatory power to account for seemingly conflicting empirical phenomena. If attentional sensitization of automatic processes is a general computational principle, the fascinating question arises whether it is possible to specifically enhance or attenuate broad classes of unconscious cognitive and emotional processes in the healthy or patient population. For instance, future studies could assess whether implicit memory traces can be differentially influenced by the nature of previously activated task representations. Similarly, it could be tested whether automatic fear responses to phobia-relevant objects (such as spiders or snakes) can be attenuated in phobic patients, when the activated task representation deemphasizes visual object recognition and/or includes a positive emotional state.

Our general experimental approach that combines a first task for inducing task sets with subsequent unconscious or conscious presentation of the critical stimulus would be an ideal tool for addressing these and related questions. The notion of attentional sensitization of unconscious cognition could also help to explain and to further empirically investigate the modulatory effects of hypnotic inductions on automatic processes. A modulation of automatic processes by hypnosis has been reliably demonstrated for the Stroop interference effect (Stroop, 1935) that depends on a conflict between task-irrelevant automatic processes of word reading and task-relevant processes of color naming (Cohen, Dunbar, & McClelland, 1990). The Stroop interference effect is abo-lished when participants receive the hypnotic suggestion that (English) color words should be conceived as meaningless character strings written in an unknown alphabet (Raz et al., 2006; Raz, Moreno-Iniguez, Martin, & Zhu, 2007). The abolishment of the Stroop interference effect by hypnotic suggestion is particularly striking because the Stroop effect is considered to be a hallmark of automatic processing. Hence, our framework could contribute to a better understanding of the attentional mechanisms underlying the effects of hypnosis in research and therapeutic settings.

## Conclusion

The implicit top-down control of unconscious processing by attentional sensitization reviewed in this article evidences the adaptability of the cognitive system in optimizing ongoing processing toward the pursuit of an intended goal: Task-relevant information is prioritized and task-irrelevant, possibly interfering influences are attenuated, both at a conscious and an unconscious level. The unconscious processing streams are thus under the control of higher-level attention to some extent. The proposed attentional sensitization mechanism operates in such a fashion as to considerably reduce the risk that unintended and not goal-related unconscious processes determine cognition and eventually influence behavior (Kiefer & Martens, 2010).

Although much progress has been made to elucidate the attentional control mechanisms of unconscious visual perception, several open issues crucially deserve further investigations:

1. If the proposed attentional sensitization model is a general theory, it should also apply to other forms of unconscious pro-cesses such as visuo-spatial and emotional processing. In particular, conscious control of unconscious emotional processing is clinically highly important because findings in this area might help to design more efficient therapeutic treatment techniques for mood and anxiety disorders.

2. In order to gain more insight in the specificity of the attentional sensitization mechanism, it is necessary to compare top-down control of subliminal priming with a variety of stimuli (e.g., verbal and pictorial stimuli) within one and the same priming paradigm. The question arises whether or not processing of subliminally presented pictures and words is boosted by the same task sets.

3. Attentional control of unconscious visual perception was mainly investigated using the induction task paradigm or by manipulating action intentions. However, as the attentional sensitization model should also be valid for control settings established with other techniques, it would be highly interesting to investigate top-down control of unconscious cognition induced, for instance, by hypnotic suggestion (Raz et al., 2006) or by subliminal task cues (Mattler, 2003; Reuss, Kiesel, Kunde, & Hommel, 2011).

4. The assumption of the attentional sensitization model that control of unconscious processes is exerted by a prefrontal top-down signal, which in turn influences the sensitivity of processing pathways in posterior brain areas, should be tested in more detail by means of fMRI and electrophysiological recording techniques.

5. Finally, formal computational modeling of the proposed attentional sensitization mechanism (see e.g., Trapp, Schroll, & Hamker, 2012) is desirable to render our theory more precise and to derive further empirically testable predictions regarding the dynamics of attentional control of conscious and unconscious visual processing in various task domains.

In conclusion, the present review described striking evidence for implicit top-down control of unconscious processing by attentional sensitization. We demonstrated that preemptive top-down control of unconscious processes coordinates the processing streams in congruency with higher-level task representations in various domains of cognition and emotion. Hence, attentional sensitization of automatic processing optimizes ongoing processing toward the pursuit of an intended goal and ensures a high degree of flexibility and adaptability of the cognitive system in unconscious visual processing.
